# Supplementary material for: Nicotinamide mononucleotide adenylyltransferase uses its NAD+ substrate-binding site to chaperone phosphorylated Tau
Source: eLife. 2020 Apr 6;9:e51859. doi: 10.7554/eLife.51859 (PMC7136026; doi:10.7554/eLife.51859)
Supplement: Supplementary file 1. [file elife-51859-supp1.docx]

表1-1 mNMNAT3数据收集以及结构精修的信息

Table 1.1Data collection and structure refinement statistics of mNMNAT3.

| Name | mNMNAT3 |
| --- | --- |
| **Data collection** |  |
| BeamLine/detector | SSRF BL17U/ ADSC Quantum 315r |
| Wavelength (Å) | 0.9791 |
| Space group | *P*2_1_ |
| Cell dimensions (Å, º) | a = 53.7, b = 80.8, c = 64.5  α= γ = 90，β = 102.2 |
| Resolution (Å)^a^ | 2.00 (2.07-2.00) |
| No. of measured reflections | 192927 |
| No. of unique reflections^a^ | 35818 (3645) |
| Redundancy^a^ | 5.4 (5.5) |
| Completeness (%)^a^ | 98.2 (100) |
| Average (I/σ)^a^ | 11.5 (5.2) |
| *R*_merge_ (%)^a,b^ | 0.102 (0.376) |
| **Refinement** |  |
| No. of reflections | 35772 |
| R_work_/R_free_^c^ | 0.1985 / 0.2415 |
| No. of non-H atoms |  |
| Protein | 3531 |
| Waters | 119 |
| Ligand | 0 |
| Average B factor [A^2^] | 45.4 |
| RMS deviations |  |
| Bond lengths (Å) | 0.009 |
| Bond angles (°) | 1.089 |
| Ramachandran plot favored (%) | 95.08 |
| Ramachandran plot allowed (%) | 3.75 |
| Ramachandran plot outliers (%) | 1.17 |

^a^Numbers in parentheses are values for the highest-resolution shell.^b^*R*_merge_ = ∑*_hkl_*∑_i_|*I*_i_ -〈*I*〉|/∑*_hkl_*∑_i_|〈*I*〉|, where I_i_ is the intensity for the *i*th measurement of an equivalent reflection with indices h, k, and l.^c^*R*_free_ was calculated with the 5% of reflections set aside randomly throughout the refinement.
