# Supplementary material for: Nicotinamide mononucleotide adenylyltransferase uses its NAD+ substrate-binding site to chaperone phosphorylated Tau
Source: eLife. 2020 Apr 6;9:e51859. doi: 10.7554/eLife.51859 (PMC7136026; doi:10.7554/eLife.51859)
Supplement: Supplementary file 2. [file elife-51859-supp2.docx]

| **Score** | **Mass(Da)** | **δ（ppm）** | **Pr 1** | **S** | **E** | **Res** | **Sequence** | **Pr 2** | **S** | **E** | **Res** | **Sequence** | **CL** | **Enz** |
| --- | --- | --- | --- | --- | --- | --- | --- | --- | --- | --- | --- | --- | --- | --- |
| 2.06E-17 | 2290.2425 | 1.066264 | mN3 | 206 | 215 | 206 | **K**ALGQGQSVK | K19 | 341 | 349 | 343 | SE**K**LDFKDR | BS^3^ | trypsin |
| 8.49E-17 | 3795.8907 | 3.010897 | mN3 | 80 | 98 | 95 | VDPWESEQAQWMETV**K**VLR | K19 | 1 (244) | 254 | 1 | **M**QTAPVPMPDLK | BS^3^ | trypsin |
| 2.71E-16 | 2859.5673 | -0.446571 | mN3 | 206 | 215 | 206 | **K**ALGQGQSVK | K19 | 354 | 370 | 369 | IGSLDNITHVPGGGN**K**K | BS^3^ | trypsin |
| 2.28E-15 | 4489.2816 | -0.05257 | mN3 | 80 | 98 | 95 | VDPWESEQAQWMETV**K**VLR | K19 | 350 | 369 | 353 | VQS**K**IGSLDNITHVPGGGNK | BS^3^ | trypsin |
| 2.95E-15 | 3101.6943 | -0.287907 | mN3 | 206 | 215 | 206 | **K**ALGQGQSVK | K19 | 354 | 372 | 370 | IGSLDNITHVPGGGNK**K**IE | BS^3^ | trypsin |
| 6.02E-14 | 3605.7965 | 0.598204 | mN3 | 80 | 98 | 95 | VDPWESEQAQWMETV**K**VLR | K19 | 341 | 349 | 347 | SEKLDF**K**DR | BS^3^ | trypsin |
| 8.81E-12 | 3605.7942 | -0.022741 | mN3 | 80 | 98 | 95 | VDPWESEQAQWMETV**K**VLR | K19 | 341 | 349 | 343 | SE**K**LDFKDR | BS^3^ | trypsin |
| 7.83E-11 | 3474.7895 | -0.062738 | mN3 | 39 | 56 | 55 | YQVIEGIISPVNDSYG**K**K | K19 | 1 (244) | 254 | 1 | **M**QTAPVPMPDLK | BS^3^ | trypsin |
| 1.13E-08 | 2480.3239 | -0.458408 | mN3 | 206 | 215 | 206 | **K**ALGQGQSVK | K19 | 1 (244) | 254 | 1 | **M**QTAPVPMPDLK | BS^3^ | trypsin |
| 2.30E-08 | 4390.2651 | 1.792606 | mN3 | 39 | 64 | 56 | YQVIEGIISPVNDSYGK**K**DLVASHHR | K19 | 1 (244) | 254 | 1 | **M**QTAPVPMPDLK | BS^3^ | trypsin |
| 2.39E-08 | 4190.2295 | -0.516917 | mN3 | 131 | 148 | 139 | LLCGADVL**K**TFQTPNLWK | K19 | 354 | 372 | 369 | IGSLDNITHVPGGGN**K**KIE | BS^3^ | trypsin |
| 2.58E-07 | 4175.125 | 0.524535 | mN3 | 80 | 98 | 95 | VDPWESEQAQWMETV**K**VLR | K19 | 354 | 370 | 369 | IGSLDNITHVPGGGN**K**K | BS^3^ | trypsin |

Δ: mass error for crosslink assignments; Pr: protein;

S, E: starting and ending amino acid residues (sequence numbers) of the cross-linked peptides, respectively;

Res: crosslinked residue (sequence number) within corresponding peptide;

CL: crosslinking reagent used; Enz: digestion enzyme used.

The crosslinked residues are bolded and underlined in the sequences.
